# Facilitator perspectives on in-person versus videoconference delivery of a remedial intervention for impaired drivers: a qualitative study

**DOI:** 10.1186/s13722-025-00626-2

**Published:** 2025-12-22

**Authors:** Chloe Docherty, Jennifer Rup, Gina Stoduto, Susan Labadia, Heulwen A. Williams, Rosely Flam-Zalcman, Tinsae Neamen, Branka Agic, Nigel Turner, Wei Wang, Christine M. Wickens

**Affiliations:** 1 Institute for Mental Health Policy Research, Centre for Addiction and Mental Health, Toronto, Canada; 2 Back on Track, Centre for Addiction and Mental Health, Toronto, Canada; 3 Education Services, Centre for Addiction and Mental Health, Toronto, Canada; 4 Dalla Lana School of Public Health, University of Toronto, Toronto, Canada; 5 Biostatistics Core, Centre for Addiction and Mental Health, Toronto, Canada; 6 Campbell Family Mental Health Research Institute, Centre for Addiction and Mental Health, Toronto, Canada; 7 Institute of Health Policy, Management and Evaluation, University of Toronto, Toronto, Canada; 8 Department of Pharmacology and Toxicology, University of Toronto, Toronto, Canada

**Keywords:** Impaired driving, Alcohol, Substance use, Remedial education, Videoconferencing, Telemental health, Telemedicine

## Abstract

**Background:**

Remedial education programs for drivers who have committed an impaired driving offence have been adopted in many jurisdictions worldwide to address impaired driving recidivism. Back on Track (BOT) is a three-part program in Ontario, Canada, which includes an 8-hour or 16-hour workshop. Although originally mandated by the Province to be delivered in-person only, a shift to online workshop delivery was required during the COVID-19 pandemic, when public health measures forbid public gathering. This study aimed to identify: (1) benefits and drawbacks for impaired driving offenders attending the program via videoconferencing technology, and; (2) potential improvements for videoconferencing-based delivery, from the facilitators’ perspective.

**Methods:**

Semi-structured interviews were conducted with ten BOT facilitators who had experience delivering the 8-hour workshop both in-person (before the pandemic) and online via videoconferencing. Interviews were conducted via Webex, were audio-recorded, transcribed, and thematically analyzed.

**Results:**

Facilitators noted that online delivery of BOT improved participant access to the program and allowed BOT providers to accommodate participants from beyond their own geographical catchment area, facilitating earlier completion of the program. However, because access to the Internet or a home computer is not universal and some participants are less familiar with videoconferencing technology, videoconferencing does not address all access issues. The ability to mute workshop discussion when online facilitated movement through curriculum and private virtual spaces could be used for one-on-one communication with any participant under the influence of alcohol or drugs. Anxiety and discomfort associated with attending an addictions treatment centre in the company of strangers was alleviated. Instead of one 8-hour in-person day, the two 4-hour online days were perceived by facilitators as more manageable and less rushed. While facilitators noted a steep learning curve in use of videoconferencing software, technology malfunctioning sometimes posed a challenge. It was also more difficult to build rapport and create connections with participants in an online setting.

**Conclusions:**

Facilitators mostly agreed that BOT participants likely benefit as much from the program online as they do in-person, and suggested that online workshops should continue because the benefits outweigh the disadvantages. Facilitators also recommended that in-person workshops be offered for those who cannot access online platforms.

**Supplementary Information:**

The online version contains supplementary material available at 10.1186/s13722-025-00626-2.

## Background

### Alcohol- and drug-impaired driving in Canada

Alcohol and drug impairment significantly increase motor vehicle collision risk, contributing to preventable fatalities and injuries worldwide [3, 24, 25, 53]. In Canada, impaired driving contributed to 18.4% of fatal collisions in 2021 [49]. The most recently available costing data indicate that Canada bore a financial burden of $20 billion resulting from impairment-related crashes in 2010 [35].

### Remedial education

Problem drinking and alcohol use disorder are frequent contributors to impaired driving behaviour. For this reason, a primary strategy to counter impaired driving involves educational programs tailored for drivers who have committed an impaired driving offence, integrating principles of brief intervention for alcohol-related issues [16, 20]. Such remedial education initiatives have demonstrated notable effectiveness in reducing alcohol consumption, associated problems, and recurrence of impaired driving offences [11, 22, 23, 54, 55].

### Back on Track (BOT)

Managed by the Centre for Addiction and Mental Health (CAMH) since 1998, Back on Track (BOT) is Ontario’s remedial education initiative designed for drivers who have committed an impaired driving offence. BOT is mandatory for all drivers convicted of impaired driving who are seeking relicensing after a period of licence suspension. The program incorporates principles of brief intervention for alcohol-related issues and comprises three main components: (1) an initial assessment to evaluate the participant’s risk of re-offending, (2) an 8-hour or more intense 16-hour workshop, depending on the results of the assessment, and (3) a follow-up interview conducted six months post-workshop completion. Workshop content includes discussion of how alcohol is processed by the body, health effects of alcohol, how alcohol and other drugs affect driving, legal consequences of and strategies to avoid impaired driving. Tools for evaluation are built into the BOT program; anonymous surveys collected before and at the end of the workshop have demonstrated improvements in attitudes related to impaired driving, affect, and self-efficacy and behavioural intentions to avoid future impaired driving behaviour [57]. Substance use (e.g., alcohol, cannabis, tobacco) and harms related to substance use (e.g., problems with mood, aggression, relationships) are measured during the assessment and 6-month follow-up interviews, and have demonstrated positive change during BOT program participation [46, 56]. The Ministry of Transportation Ontario conducted its own assessment of BOT and reported a 35% reduction in the three-year cumulative incidence of recidivism following introduction of the program [22].

### Online delivery of psychotherapeutic interventions

In recent years, with widespread expansion, access to, and use of telecommunications technology (e.g., texting, email, Internet, smartphones), multiple innovative approaches have been developed for supplementing in-person psychotherapeutic interventions or providing mental health resources for those not otherwise able to access mental health services [28, 52]. Moreover, the proliferation of high-quality videoconferencing technology for computers and smartphones has augmented the feasibility and acceptance of substituting online for face-to-face delivery of psychotherapeutic treatment [30]. Prior to the COVID-19 crisis, declared a pandemic by the World Health Organization on 11 March 2020, there was an emergence of literature reviews citing videoconferencing-based delivery of psychotherapeutic interventions as equally effective as traditional face-to-face interventions [4, 5, 7, 9]. Some initial concerns about distance delivery, such as a therapist struggling to foster a therapeutic relationship with their patient, were deemed unwarranted ([17, 41]; see [31] for dissenting conclusions). Advantages of this new delivery mode were identified: increased accessibility, reduced costs, and patient disinhibition (i.e., more free therapeutic expression), encouraging self-reflection and ownership of the therapeutic process [28, 38, 47]. Still, some concerns remained, including obstacles posed by the new medium in reading a patient’s non-verbal body language or in the therapist being able to appropriately intervene should a patient be in crisis, both of which had potentially dire consequences for patient well-being or safety [38, 39, 47]. These pre-pandemic reviews also highlighted important gaps in the existing literature, including a limited number of randomized controlled trials and minimal focus on treatment of alcohol use problems specifically [2, 21].

The COVID-19 pandemic forced a sudden shift to online delivery of mental healthcare services, including treatment for alcohol and drug use. With this shift came a new wave of empirical studies of the effectiveness of online psychotherapeutic interventions. Updated reviews and meta-analyses that incorporated this new wave of studies provided further evidence of similar levels of effectiveness for in-person and online delivery of alcohol and drug use treatment [13, 15, 18, 19, 26, 32, 40]. Nonetheless, gaps in the literature remained, with reviews continuing to call for high-quality randomized controlled trials of specific addictions in addition to other needs, including exploration of the therapist experience, therapist training in online interventions, and cost savings of the new delivery medium, to name a few [13, 18, 19, 34, 50, 51].

### Piloting distance delivery of BOT in 2018

Prior to the COVID-19 pandemic, BOT workshops were exclusively conducted in-person. This face-to-face approach to program delivery aligned with conventional practices for delivery of individual or group psychotherapeutic interventions. However, challenges in accessing BOT in remote areas of the province were recognized as an important equity issue for drivers living outside of major cities. Reviews and meta-analyses of videoconferencing-based psychotherapeutic interventions had begun to emerge, concluding that this delivery modality was equally effective as traditional face-to-face therapies [4, 5, 7]. However, to our knowledge, none of the existing studies focused on education or treatment programs designed specifically for individuals who had been convicted of an impaired driving offence. Therefore, in 2018, a preliminary evaluation of simulated videoconferencing delivery of the BOT program was undertaken to anticipate potential issues associated with this delivery modality [29]. Twenty BOT participants volunteered and were randomly assigned to either an in-person 8-hour workshop or a workshop delivered via videoconference. Participants assigned to videoconferencing accessed the workshop through CAMH’s Ontario Telemedicine Network (OTN), utilizing interactive video technology. While BOT facilitators and in-person participants remained in the main room, videoconference participants were situated in a separate room where they viewed a live feed of the main room on a television monitor. Simultaneously, a video feed of the videoconference participants was transmitted back to the BOT facilitators in the main room.

Upon completion of the pilot study, participants in both delivery modes showed enhanced attitudes toward impaired driving, with minimal differences observed between videoconferencing and in-person groups. However, videoconference participants reported lower levels of engagement and satisfaction with the program compared to their in-person counterparts, and rated the curriculum as less clearly presented. Challenges included issues with sound and video quality, particularly during the presentation of in-class videos. In debriefing following the pilot study, facilitators involved with the exercise expressed concerns about being able to detect substance use by videoconference participants during the workshop, which would be in violation of BOT program requirements and would typically result in the participant being expelled from the program.

### Purpose of current research

As was the case for many providers of substance use treatment and education, delivery of the BOT program was forced to transition online using videoconferencing technology during the COVID-19 pandemic. Unlike the OTN tested in the 2018 pilot study, widely adopted videoconferencing platforms used during the pandemic, including Zoom, Teams, and Webex, could be accessed via personal computers or smartphones, enabling each participant to use their own camera and monitor, and to share content from their devices with the group. This advancement in technology limited generalizability of the pilot study’s findings to present day. Therefore, a two-study project was initiated to determine how the shift to online delivery had affected the BOT program, and to identify potential solutions to any challenges encountered during the shift. The first study was a randomized controlled trial examining how the transition to online delivery of BOT impacted client experience of the program as well as program outcomes (see [58]). The second study, reported here, was a qualitative interview study examining how the shift online impacted facilitators’ perceptions of program delivery.

### Research questions

Qualitative research methodologies enable a more nuanced understanding of how individuals experience phenomena than is possible through quantitative methodologies. This one-on-one interview study with facilitators of the BOT 8-hour workshop aimed to inform two key research questions:

1. What are the benefits and drawbacks of delivering via videoconferencing technology a remedial education program for drivers who have committed an impaired driving offence?;
2. What improvements, if any, could be made to videoconferencing-based delivery of the BOT program?

In addressing these research questions, which focused on improving the BOT program specifically, the current study addressed two understudied gaps in the existing literature: (1) online treatment of alcohol use, as opposed to other substances of abuse, and; (2) exploration of the therapist experience with online treatment. Moreover, because the study examined individuals convicted of an impaired driving offence attending a remedial education program, it explored what remains, to our knowledge, an unstudied population and form of intervention with respect to distance delivery of psychotherapeutic treatment.

## Methods

To address these research questions, semi-structured interviews were conducted with BOT facilitators who had experience delivering the program both in-person (before the pandemic) and online via videoconferencing technology such as Webex, Teams, or Zoom. Methods and results of this interview study are reported using the Consolidated Criteria for Reporting Qualitative Research (COREQ) checklist (see Additional File 1; [48]), which is designed to enhance transparency, improve quality, and facilitate critical appraisal of qualitative research.

### Recruitment and data collection

Purposive sampling [33] was used to select facilitators to be interviewed, with attention paid to including individuals who facilitate BOT in different regions across Ontario. Most BOT facilitators are female, but an effort was made to recruit male facilitators. BOT management provided to the research team a list of facilitators who met inclusion criteria and sent an email to these facilitators inviting them to contact research personnel by email about participating in a study to understand how facilitating a BOT workshop online via videoconferencing may differ from facilitating in-person. Facilitators who agreed to be interviewed were compensated with a $25 e-gift card. Recruitment for facilitator interviews continued until saturation in the ongoing interviews was reached.

### Inclusion criteria

To be eligible for participation, facilitators had to have experience leading both the in-person and videoconference-based 8-hour BOT workshop. They also had to have a smart-phone, tablet, or computer with Internet access in order to participate in an online interview.

### Materials

The semi-structured interview guide posed six open-ended scripted questions covering the following topics: experiences facilitating BOT online and in-person, advantages and disadvantages of delivering BOT online versus in-person, whether online participants benefit as much as in-person participants, and recommendations for an improved online program experience. Recognizing that remedial education delivered in a group setting may be characterized by unique features and challenges (e.g., focus on avoiding impaired driving, mandated attendance for licence reinstatement) that are not shared by other more typical therapeutic settings, scripted questions were deliberately broad and high-level, allowing facilitators to identify the most salient issues related to program delivery modality. Unscripted follow-up questions were sometimes asked to allow participants to elaborate on or clarify their previous statements. The interview guide is provided in Additional File 2.

### Procedures for interview

Potential participants who contacted the research team were asked via email to confirm that they met the eligibility criteria. Once eligibility was confirmed, the interview was scheduled. Participants were instructed to find a quiet place where they could be alone when being interviewed. Interviews were expected to take up to 30 min and were conducted via Webex between December 7th and 29th, 2022. Field notes by the interviewer were not needed before or after interviewing. The interviews were audio recorded and fully transcribed. All identifying information was stripped from transcripts. Interviews were conducted by the first author (CD) who identifies as a female and woman, holds a Bachelor’s degree, and works in a research environment. The interviewer met with the senior author (CMW; who identifies as a female and woman, holds a PhD, and works in a research environment) to discuss interviewing strategy and techniques, reviewed training videos and published articles on interviewing strategy, and conducted practice interviews with lab personnel prior to initiating interviews with study participants. No existing relationship existed between the participants and the interviewer before participants reached out to the interviewer to confirm eligibility and schedule the interview. The first and senior authors conduct ongoing evaluations of the BOT program, but rarely interact with program facilitators. Beyond what was contained in the recruitment email, no biases, assumptions, and goals for the research held by the research team were shared with participants.

### Analysis

Data were managed and analyzed using NVivo 12.0 software. Memos were written by the interviewer and primary analyst (CD) throughout the analysis process to track emerging insights. A preliminary codebook was developed by the primary and secondary analyst (second author, JR, who identifies as a female and woman, holds a Master’s degree, and works in a research environment), based on the research questions, memos, and review of three interview transcripts. Refinement of the coding structure was achieved through an iterative process, with the primary and secondary analysts coding interview transcripts independently, then meeting to discuss discrepancies and further refinement of the structure. This process was repeated until the analysts had fully coded all transcripts.

Analysts adopted an essentialist/realist epistemology. Participants’ real experiences and perspectives were reported and summarized, and the meaning of their reality was examined to develop and interpret codes and themes. Thematic analysis was conducted following Braun and Clarke’s [10] approach. Analysts coded semantically, presenting the content of the data as communicated by the respondent, and adopted an experiential orientation, recognizing that the insights shared to answer the research questions were informed by the participants’ experience as program facilitators. An inductive approach was used to connect responses across the interviews, and then themes and subthemes were identified based on open coding of the transcripts. Regular meetings were held with the study team to discuss emerging themes, and the codes and themes were subsequently refined. Themes, subthemes, and descriptions were further revised until they reflected the dataset. Similar to the approach adopted for the interview guide, this inductive approach to data analyses was selected because remedial education in a group setting may have unique characteristics that limit the applicability of existing research. The inductive approach provided flexibility to identify patterns that might deviate from those seen with other more typical psychotherapeutic interventions. See Byrne [14] for an applied example of this methodological orientation and theory.

## Results

### Screening and final sample

Thirty-eight BOT facilitators were contacted by BOT management, inviting them to take part in the interview study. Seventeen facilitators contacted study personnel expressing their interest to participate. Purposive sampling, with the goal of including both male and female facilitators, as well as facilitators from all regions of the province, led to the selection of 11 facilitators who were screened for eligibility. One of the 11 screened facilitators was ineligible due to not having previously facilitated the 8-hour workshop. Of the ten facilitators interviewed, three were male and seven were female. Of the participants that were interviewed, nine were from Southern Ontario and one was from Northern Ontario, defined as any BOT provider north of or including Bracebridge/Parry Sound. Interviews ranged in length from about 7 to 45 min, with interview length mean and median at approximately 24 and 21 min, respectively. A table outlining each participant’s sex, region, and interview length is provided in Additional File 3.

### Increased program accessibility and ability to accommodate more clients

Facilitators spoke about how eliminating the need to drive to a physical location for a primarily non-driving population (due to licence suspension) improved access to the BOT program and made participation more convenient. Distance, weather, and absence of transportation options were no longer challenges for those participants attending the program via videoconference.

*[T]he weather didn’t play a part into um doing the workshop…people didn’t have to worry about traveling on those roads…the icy roads. So we didn’t have to do any kind of cancellation or rescheduling*,* so that’s always handy…all of our clients*,* you know*,* they don’t have a driver’s licence so they do have to depend on other people to take them.* – Participant 5

*[O]ur attachment geographic area is big so when they were coming in in-person*,* they were driving sometimes like hours … Right? Unlike Toronto*,* it’s not easy to just jump on a y’know subway or Uber or anything like that. So*,* a lot of them were grateful for that.* – Participant 10

Some facilitators mentioned that online workshops had eliminated geographical barriers, allowing BOT facilitators to service people across Ontario as opposed to just those in their catchment area. As a result, facilitators speculated that removal of these geographical barriers was enabling participants to complete their program requirements sooner.

*It allowed us to expand and allow the participants to have more options with regards to getting their*,* um*,* their steps to the Back on Track program done quicker because they can join [any] group.* – Participant 10

### Easier to maintain a respectful and supportive learning environment

Facilitators believed that online workshops gave them more control over the learning environment and enabled them to more easily create a respectful and supportive space for learning. Muting participants’ microphones could be used to bring class discussion to a close when it was time to move on to the next curriculum component. On rare occasions, participants are excused from the program because they appear to be under the influence of alcohol or drugs, in contravention of program requirements. Virtual delivery of the workshop enabled facilitators to easily transition to a private virtual space to communicate one-on-one with any participant under the influence of alcohol or drugs, reducing stress for both the facilitator and participant, and minimizing distraction for other workshop participants.

*[I]t’s easier to cut conversations. When you’re in-person you have to have a really strong facilitator ability to do that. But with the power of a mute button*,* imagine how quickly a conversation is like cut right off the bat when it’s virtual*,* right?* – Participant 1

*In some ways*,* my personal sense of safety feels different doing it virtually versus in-person… If I was in-person turning someone away*,* if I suspected that they were going to be*,* um*,* having a real response to that… and I was telling someone that they were going to not pass the workshop. It’s easier to do that across the screen still respectfully*,* privately*,* everything*,* but they’re not with me… they may have a reaction*,* but it’s going to be felt differently by me across the screen than when they are in the boardroom with me.* – Participant 7

Facilitators mentioned that many participants felt anxiety when attending the workshop in-person, experiencing discomfort because they were attending the workshop at an addiction treatment centre, in the company of strangers. Facilitators speculated that online workshops, being experienced from the comfort of one’s own home, alleviated the anxiety and discomfort otherwise felt by some participants.

*Doing it online*,* they can actually be in their own house. I find them to be a little bit more relaxed*,* because they are in their own environment rather than coming to a strange facility to do it… Um*,* surrounded by a bunch of strangers. Whereas online even though they are surrounded by a bunch of strangers in a box on their computer screen… I don’t think they have the anxiety when they’re at home as what they would in the classroom scenario.* – Participant 4

*I wondered sometimes what their experience was*,* of coming to an addiction agency to attend this workshop… The feeling of shame like it was evident sometimes when people were in the group*,* you know*,* we tried to create as positive as human as non-judgmental a space to say like*,* this is learning*,* this is helpful. And yet*,* I think coming to the building. I think it was hard for a lot of people.* – Participant 7

While in-person education workshops consisted of an 8-hour single-day session, videoconferencing-based workshops consisted of 4-hour sessions on two separate days. Facilitators believed that splitting the online workshops across two days increased manageability of the workshop content and provided more flexibility. Facilitators reported feeling less rushed and believed that information retention and engagement had improved.

*It feels much more manageable to be like*,* I have to get through this much today*,* versus I got to get through everything today. When there is something unexpected that comes up in a workshop. When there is a client who is um*,* in distress*,* someone has been using substances*,* someone who is having- really disengaged*,* cameras turned off*,* falling asleep… Usually like*,* that takes some time away from the group. I think*,* in a 2-day virtual workshop*,* it feels like there’s just more buffer.* – Participant 7

*I found that in-person*,* you know*,* after lunch*,* everybody’s got a belly full of food*,* and they could use a nap [laughs]. And plus you’re just sitting there*,* like*,* giving information*,* information*,* information and it’s hard. To sit for eight hours and just be involved and and take in that information as the participant. Right? So*,* I found that there was always this lull in the afternoon that it was kind of like*,* you know*,* dragging people through it. And I and I don’t feel like we have that now.* – Participant 3

### Potential challenges with technology

Problems and glitches with videoconferencing technology, and staff or participants not knowing how to address these operational issues, were identified as a challenge posed by videoconferencing-based workshops.

*I’m always*,* you know*,* mindful okay how is my own lighting? Am I getting shadowy? Am I breaking up? Am I freezing? My co-facilitator*,* we rely on each other*,* to say*,* oh*,* [name]*,* you*,* you broke up a bit there and we try to repeat things. Um*,* sometimes just hearing all the people can be a challenge and we try to address it*,* but at the end of the day*,* it’s across the screen*,* and you’re limited by the technology that you have*,* and the technology that they have.* – Participant 7

Most facilitators commented on the initial steep learning curve with the various online platforms used to run the videoconferencing-based workshops. Although each service provider used different videoconferencing software, these comments were made by users of several different software packages.

*So*,* at the beginning*,* like*,* getting to the technology and all the security and all the information and stuff. But now*,* I’m pretty comfortable. Even our participants. They’re great. Because some have more technology information*,* or*,* smarts I guess than I do*,* so they can say they can share that with other participants ‘oh try this*,* or y’know try this*,* or*,* you know*,* do that.’ So*,* it was really*,* I think that was the biggest challenge was being more confident to be able to offer that service.* – Participant 10

Facilitators believed that online workshops may not be equitable for all clients; some clients do not have access to the Internet or a computer at home, and others are less familiar with use of videoconferencing technology.

*Some of the population and I shouldn’t kind of stereotype*,* but*,* the older population that’s not quite as familiar with technology*,* I think has a lot of anxieties with doing it [the workshop] virtually.* – Participant 5

### Difficult to build connections/rapport and coordinate interactions

Facilitators spoke about the connections that develop among participants, and between facilitators and participants, during a workshop as common experiences and ideas are shared. Facilitators noted that it is more difficult to create these connections and build this rapport with and between clients in an online setting because interactions are more limited in the virtual environment.

*One of the things that’s not great about virtual is the beauty of connecting with other people when you walk into a room and finally have people that you kind of go. Oh*,* yeah. Like you get it. You’ve been through this without judgment or pain or consequence. Right? And connecting with people on that front*,* that’s doable in-person.* – Participant 1

### Challenges with observing client body language and managing distractions

Facilitators noted that prior to initiating videoconferencing-based delivery of the BOT workshop, they had concerns about how this new medium might interfere with their ability to read body language, which they felt was essential to detect substance use (forbidden while attending the program). However, facilitators commented that although more challenging in the virtual medium, detection of substance use by participants could still be done.

*It would be easier for somebody to get away with substance use while they’re in the course. Can’t smell booze on someone’s breath on Zoom… it is more challenging to know if somebody is drinking or using… And this isn’t good right? And so we have had to remove people and of course*,* they get pissed off and they claim that they aren’t and whatever else and*,* like*,* how are we going to prove it? … So*,* there is more challenge in that.* – Participant 3

Likewise, facilitators expressed concern about their ability to detect participant distraction or participants who were not participating in the workshop when delivered virtually. There are more sources for distraction when a participant is attending the workshop from home, and it is more difficult for facilitators to ensure that participants are paying attention. However, facilitators grew more confident in their ability to detect distraction and lack of engagement.

*There is the distractions in your own home. Uh*,* it can make it hard for some people to engage. But I feel on the whole most people engage very well… it’s easier to know if somebody’s really engaged [if] they’re sitting in front of you in a classroom*,* than if they’re sitting at home*,* but after a while you do learn*,* we’ve learned what those little signs or signals are.* – Participant 3

### Coordinated and collaborative training would improve online delivery of the BOT program

Facilitators felt that they would benefit from logistical training for the online platform, as well as updated content training on the material. Sharing basic training on use of the videoconferencing technology with participants ahead of the workshop was also strongly recommended.

*I think right now a lot of the agencies*,* we know we all use different technology*,* right? So*,* knowing what is kind of expected with the program*,* in terms of the technology… I think the more that you ask participants to do extra little things virtually*,* the more anxiety they get. Doing something new*,* like*,* learn how to get into a breakout room and get back out of a breakout room and things like that. So*,* having more training in terms of what the actual program is capable of and what is expected from us… virtually like*,* what our role is virtually.* – Participant 5

*I think it is important …prior to the group starting*,* that the participants get kind of a detailed explanation on how to use the technology and what’s expected of them. I think it needs to be laid out pretty clearly before the group starts… I usually send out a memo or a letter that says*,* you know*,* this is how to use Teams. This is what’s expected and as things come up*,* I may add to that.* – Participant 5

Facilitators recommended more communication and collaboration among their fellow facilitators regarding successes and challenges of delivering the program online, suggesting that such discussions would help to create a more standardized process that incorporated the best strategies.

*I think any opportunity for which we can come together as providers to talk about… what are some wins? And what are some downfalls?…And how do we manage our downfalls? Opportunity for us to learn together is not something that happens*,* we need that.* – Participant 1

### Facilitator conclusions concerning the value of delivering the BOT program online

For the most part, facilitators agreed that BOT participants likely benefit as much from the program online as they do in-person. Facilitators tended to acknowledge benefits and drawbacks of both in-person and videoconferencing-based program delivery, but felt that overall value of the program to participants was likely equivalent across the two delivery methods.

*We were apprehensive about it. We thought this is going to be brutal. This isn’t going to work. I don’t know how this is possibly going to be effective*,* but we’ve actually found it that it’s been a lot more convenient. And I think the effectiveness is still there.”* – Participant 3

*A lot of my initial concerns have not been realized. One of my major concerns was client engagement. I worried that*,* knowing people were often coming to Back on Track with a very low expectation*,* and sometimes a fairly negative expectation of what was to unfold*,* I worried that somehow the screen wouldn’t be enough to engage people. That fear hasn’t been realized because over time*,* I think*,* we have been able to provide the same workshop*,* just in a different format.* – Participant 7

Many facilitators suggested that online workshops should continue to be offered moving forward, reasoning that their benefits outweigh their drawbacks. Despite believing that the impact of the BOT program for participants was equivalent across delivery modes, facilitators also recommended that in-person workshops continue to be offered for those participants who cannot access the online delivery mode. A hybrid option was also suggested where the participant physically attends a BOT location but participates online via videoconferencing technology with the assistance of BOT personnel.

*If we have somebody who’s very technical with it [videoconferencing]*,* it’s*,* uh*,* absolutely … no problem whatsoever. I do believe that offering the services and continuing to offer them online in a hybrid kind of way would be extremely beneficial. Especially for those people who have not told anybody about the impaired driving*,* live in remote areas*,* no transportation*,* things like that.* – Participant 6

## Discussion

Based on one-on-one semi-structured interviews with facilitators of Ontario’s BOT remedial education program for drivers who have committed an impaired driving offence, facilitators expressed their belief that the program was equally effective regardless of in-person or videoconferencing-based delivery. These beliefs were generally supported by the randomized controlled trial contrasting clients who attended BOT in-person versus via videoconferencing (Study 1; [58]), which found an absence of significant differences in client satisfaction, clarity of presentation, learner engagement, and positive change in attitudes, negative affect, self-efficacy, and behavioural intentions related to impaired driving following workshop participation. Facilitators identified both drawbacks and benefits of program delivery by videoconferencing versus in-person, but recommended continued offering of the program through the videoconferencing medium.

### Drawbacks of delivering BOT via videoconferencing

Among the drawbacks of delivering BOT via videoconferencing, and consistent with research of videoconferencing-based psychotherapy [4, 6, 8], BOT program facilitators cited technical issues with the videoconferencing platform and lack of familiarity with the platform among select participants. Lack of access to technology, including access to reliable and secure Internet service, were identified as other important barriers to videoconferencing-based program delivery. Facilitators also noted that in-person interactions between facilitators and participants, and importantly shared experiences and interactions among participants, particularly during program breaks, serve to build connections and rapport in the group. There is much evidence in the scientific literature about the importance of therapeutic alliance (i.e., the relationship between a therapist and a client) and cohesion (i.e., the relationship among group members) to treatment outcomes of group therapy [1, 12]. When delivered through videoconferencing, facilitators acknowledged that virtual workshops do not afford as many opportunities for interactions and sharing of experiences that help to build alliance and cohesion, resulting in a different feel to the educational workshop. Facilitators also noted challenges in detecting distracted participants, but felt this was being overcome as facilitators gained more experience with the online medium. Facilitators acknowledged that substance use, which is not permitted within 24 h of any BOT program component, was more challenging to detect without cues exclusive to the in-person environment (e.g., scent of substances); but, facilitators also felt that their training and experience as substance use counsellors still allowed them to detect substance use virtually.

### Benefits of delivering BOT via videoconferencing

Facilitators also identified several benefits to videoconferencing-based delivery. Specifically, they noted that virtual delivery made the program more accessible for those clients living in areas of the province with limited public transit or in remote areas requiring long distance travel to a BOT provider location. This is a recognized benefit of all telehealth services [28, 52] and should not be undervalued, particularly given that in 2022 about one third of Canadians who met diagnostic criteria for a mood, anxiety, or substance use disorder in the past year and reported needing counselling or psychotherapy services also reported partially or fully unmet needs for this mental health treatment [45]. Facilitators commented that virtual delivery allowed participants to attend workshops offered by providers in different catchment areas and speculated that this allowed some participants to complete the workshop component of their program sooner than if they were forced to wait for an available workshop in their own catchment area. From a program management perspective, this ability to accommodate clients across catchment areas helps to ensure that clients can complete the BOT program within 12 months, which is mandated by the Province. For those clients who have a substance use disorder and are motivated to pursue health behaviour change (e.g., see Prochaska’s [36] discussion of the transtheoretical model, and stages of, behaviour change), more rapid access to an intervention, at the time that clients are motivated to change, improves engagement with treatment and probability of reduced substance use and related harms [44, 59].

The ability to mute workshop discussion when online facilitated movement through curriculum and private virtual spaces could be used for one-on-one communication with any participant under the influence of alcohol or drugs. Facilitators also believed that some participants were more at ease in the virtual workshop, some of whom may have felt uncomfortable or stigmatized by physically attending an addiction treatment centre, where many BOT workshops are hosted.

Finally, facilitators pointed out that videoconferencing-based workshops were spread across two days, instead of using the single 8-hour session format adopted by the in-person delivery model. Facilitators described feeling less rushed in their delivery of the curriculum in the two-day format and believed that it may give participants an opportunity to better absorb what they were learning. Existing research from education and pedagogy suggests that the adoption of more numerous but shorter learning sessions in lieu of a few long sessions, known as instructional chunking, improves learning engagement and student motivation, produces higher attention and concentration, and ultimately results in better learning [27].

### Recommendations for videoconferencing-based delivery of BOT

Facilitators provided a number of recommendations for delivery of BOT as it moves forward post-pandemic. Overall, facilitators believed that the benefits of videoconferencing-based program delivery outweighed its drawbacks and, therefore, BOT should continue to be offered this way. However, facilitators acknowledged that accessibility posed a challenge and recommended either limited continuation of in-person delivery or a hybrid option where a participant could travel to a BOT location and attend an online workshop with local BOT personnel nearby to assist with technical difficulties if needed. In remote locations, it might also be possible for clients to access the online program through a community centre, library, or other civic service centre. Facilitators also acknowledged that there is room to improve videoconferencing-based delivery of BOT. They cited a steep learning curve in how to optimally use the online platform and to overcome challenges such as participant distraction and learner engagement. They recommended that the BOT management team provide opportunities for facilitators to come together and share successful strategies using this new delivery medium with others, and that standardized training and procedures be developed and shared with facilitators across the province.

Although comfortable relying on their training as substance use counsellors, facilitators acknowledged recognition of substance use by participants as more challenging with videoconferencing-based delivery. Advancements in technology eventually provide a solution to this challenge. Multiple companies are currently developing mobile applications for detection of cognitive and motor impairment, including impairment associated with substance use (e.g., [37, 43]). As these mobile applications become more available and accessible, and if recognized standards are developed for determination of impairment based on this technology, perhaps these applications can be downloaded and used by BOT clients at the request of facilitators who suspect recent substance use by the client. This type of impairment test could provide an objective measure of impairment that may elicit less objection from the client than a subjective assessment of impairment being made by a facilitator through a videoconferencing platform.

### Strengths and limitations

A primary strength of the current study is that it expanded the existing literature on psychotherapeutic interventions delivered through an online medium, exploring a remedial education program designed specifically for individuals convicted of an impaired driving offence and mandated to attend for licence reinstatement. In doing so, it also addressed two understudied topics in that existing literature: treatment of alcohol use, as opposed to other substances of abuse, and the experience of online treatment from the therapist’s perspective.

It should also be noted that while the current study includes findings that inform online delivery of a psychotherapeutic intervention, select findings might also be relevant to other forms of online education, including those that support other forms of health behaviour change (e.g., diabetes management, nutrition and dietetics, exercise physiology). Facilitators’ insights concerning improved access to treatment through online program delivery, as well as the value of presenting curriculum in more numerous but shorter sessions (i.e., instructional chunking), would benefit other health programs considering online delivery.

The current study had a number of limitations that must be considered. The study included interviews with ten facilitators of the BOT program; this is an admittedly small sample, which may call into question the robustness of the identified themes. Repetition in facilitators’ responses, as judged by the interviewer, suggested that saturation had been reached; however, future research that builds on the current findings through more detailed questions and with a larger sample would allow for replication. The study was also conducted not long after BOT shifted to online program delivery, and therefore facilitators had much more experience with in-person than online delivery. Facilitators participating in a replication study would be more experienced with online delivery and may have even more insights to share. There was also some indication that a more theory-informed approach to question development and analysis would be beneficial, despite the differences between remedial education and other more typical therapeutic settings; for example, facilitators noted an absence of opportunities for rapport-building between facilitators and participants and among participants themselves, which is consistent with treatment approaches that emphasize the importance of therapeutic alliance and cohesion [1, 12].

Another limitation was that the current study focused on the facilitator perspective, to the exclusion of BOT participants. Facilitators sometimes shared their perspective of how online delivery may be impacting participants’ experience of the BOT program, but such perceptions should be confirmed by participants themselves. A randomized controlled trial, conducted at the same time as the current study, compared participants attending the workshop in-person versus via videoconferencing in terms of program outcomes and found high levels of client satisfaction, perceived clarity of presentation, and learner engagement regardless of program delivery method [58]. This trial’s findings inform our understanding of the participant experience of online program delivery. However, one-on-one interviews with BOT participants would have complemented well both the randomized controlled trial and the current interview study with BOT facilitators; unfortunately, due to timeline and resource limitations, an interview study with BOT participants could not be incorporated into the current project and remains an aim for future research.

Although the research met its primary objective to better understand how the shift to online delivery affected the BOT program and address any challenges encountered during the shift, there are limitations to the generalizability of the current study’s findings even to the BOT program. As the current study asked facilitators to reflect on their experiences leading the 8-hour BOT workshop, the study findings may not generalize to the 16-hour workshop which is run for clients with higher risk of recidivism. It is possible that facilitators may have shared different insights had they been asked about their experience delivering the program virtually to participants with more severe substance use problems, although participants in the 16-hour workshop also report high levels of program satisfaction [42]. Interviews with facilitators who deliver the more intense workshop for higher-risk clients should also be conducted.

Finally, although recommendations provided above for videoconferencing-based delivery of BOT may be helpful for other remedial education programs for impaired driving offenders, programs vary in structure and content from one jurisdiction to another. Therefore, practitioners are advised to conduct their own evaluations when adapting these insights to other approaches to remedial programming or other contexts.

## Conclusions

From the perspective of facilitators of a remedial education program for drivers who have committed an impaired driving offence, videoconferencing is a viable delivery method. Although challenges and drawbacks must be acknowledged, many of these can be overcome and are outweighed by the many benefits associated with virtual program delivery.

## Supplementary Information

Below is the link to the electronic supplementary material.

Supplementary Material 1: Additional File 1 (Additional File 1.docx) contains Consolidated Criteria for Reporting Qualitative Research (COREQ) checklist.

Supplementary Material 2: Additional File 2 (Additional File 2.docx) contains the study interview guide.

Supplementary Material 3: Additional File 3 (Additional File 3.docx) contains each participant’s sex, region in the province, and interview length.

## Data Availability

The anonymized interview transcripts generated and analyzed during the current study are available from the corresponding author on reasonable request.
