# Supplementary material for: Facilitator perspectives on in-person versus videoconference delivery of a remedial intervention for impaired drivers: a qualitative study
Source: Addict Sci Clin Pract. 2025 Dec 22;21:5. doi: 10.1186/s13722-025-00626-2 (PMC12805713; doi:10.1186/s13722-025-00626-2)
Supplement: Supplementary file 2 — Supplementary Material 2: Additional File 2 (Additional File 2.docx) contains the study interview guide. [file 13722_2025_626_MOESM2_ESM.docx]

**Additional File 2**

**Back on Track Facilitator Interview Guide**

The following questions ask you about your experience with the Back on Track Education Workshop participation.

We are interested in your perceptions and insights as a Facilitator in dealing with videoconference technology participation clients.

1. How would you describe your experience facilitating the Education Workshop with a group participating through videoconferencing technology?
2. How would you describe your experience facilitating the Education Workshop with a group participating in-person?
3. Do you see any beneficial aspects to providing the Education Workshop through videoconferencing technology?
4. Do you see any negative aspects to providing the Education Workshop through videoconferencing technology?
5. Do you think videoconferencing technology participants benefit as much from the program as those who participate in person?
6. If videoconferencing technology is integrated into BOT on an ongoing basis, is there anything that you would recommend to improve participant engagement in the program, and make the program effective for them, such as specific training for facilitators?
