# Supplementary material for: Facilitator perspectives on in-person versus videoconference delivery of a remedial intervention for impaired drivers: a qualitative study
Source: Addict Sci Clin Pract. 2025 Dec 22;21:5. doi: 10.1186/s13722-025-00626-2 (PMC12805713; doi:10.1186/s13722-025-00626-2)
Supplement: Supplementary file 3 — Supplementary Material 3: Additional File 3 (Additional File 3.docx) contains each participant’s sex, region in the province, and interview length. [file 13722_2025_626_MOESM3_ESM.docx]

**Additional File 3**

| **Participant Sex, Region in the Province, and Interview Length** | | | |
| --- | --- | --- | --- |
| **Participant #** | **Sex (F or M)** | **Region (N or S)** | **Interview Length (mm:ss)** |
| 1 | F | S | 26:56 |
| 2 | F | S | 12:49 |
| 3 | M | S | 38.:58 |
| 4 | M | S | 06:53 |
| 5 | F | S | 16:09 |
| 6 | F | S | 19:47 |
| 7 | F | S | 45:23 |
| 8 | F | S | 34:02 |
| 9 | M | S | 15:06 |
| 10 | F | N | 20:50 |
| *Notes:* F = female, M = male, N = Northern Ontario, defined as any BOT provider north of or including Bracebridge/Parry Sound, S = Southern Ontario, defined as any BOT provider south of Bracebridge/Parry Sound, mm:ss = minutes and seconds. | | | |
